# A population-based registry study on psoriasis-associated burden of disease in Finland

**DOI:** 10.3389/fmed.2025.1605100

**Published:** 2025-07-22

**Authors:** Liisa Ukkola-Vuoti, Anton Klåvus, Iiro Toppila, Jenni Hällfors, Lauri Veijalainen, Tarja Mälkönen

**Affiliations:** ^1^Medaffcon Oy, Espoo, Finland; ^2^UCB Pharma Oy Finland, Espoo, Finland; ^3^Skin and Allergy Hospital, Helsinki University Hospital, Helsinki, Finland

**Keywords:** registry, epidemiology, treatment, sick leaves, healthcare resource utilization, treatment switch, disease burden, psoriasis

## Abstract

**Introduction:**

Despite advancements in treatment, unmet needs persist for patients with plaque psoriasis (PSO). This study characterized PSO patients who were initiating or switching biologic medications, with or without prior biologic use, and highlighted the unmet needs due to medication switches and concomitant treatments. The impact of biologic therapy initiation was assessed through changes in healthcare resource utilization (HCRU), sick leave, and disability pensions.

**Methods:**

This study utilized electronic healthcare data of adult patients with PSO and reimbursed biologic medication purchases made between 2013 and 2021 in Finland. Patients were followed from the day of first biologic treatment purchase (first biological cohort) or switch (switchers) until 2022 or death/loss of follow-up.

**Results:**

A total of 2,437 patients with PSO and biologic medication purchases were investigated. Of this total, 14.2% (*n* = 345) were switchers, and 85.8% (*n* = 2,092) comprised the first biological cohort. Among the first biological cohort, 12.5% [95% confidence interval (CI): 11.1, 13.9] had switched to other biologic medications 1 year after initiation. Work absences started to accumulate before the initiation of the first medication in the first biological cohort, followed by a subsequent decrease, while the accumulation remained modest and linear in shape among the switchers. The proportion of patients aged <65 on disability pension was higher among the switchers compared to the first biological cohort (7.8% (*n* = 27) and 6.6% (*n* = 138), respectively). A total of 86 first biological patients (4.1%) and 11 (3.2%) switchers were receiving disability pension before the biologic treatment was initiated. The number of all-cause secondary healthcare outpatient contacts per year (11.1 vs. 7.4 per patient; *p* < 0.001) and disease-related inpatient days (0.46 vs. 0.16 per patient; *p* < 0.001) was lower 1 year after the initiation of biologic treatment in the first biological cohort compared to the time before biologic treatment. The decrease in the disease-related any-type contact cost per year for the first biological cohort was significant, from €2,098 (95% CI: 1,975, 2,221) to €1,094 (95% CI: 1,012, 1,176; *p* < 0.001). No significant reduction was observed in the HCRU of switchers.

**Discussion:**

This study highlights the need for timely treatment and underscores the significant unmet needs among patients with PSO. Further studies are needed to evaluate the overall benefits of early utilization of highly effective treatments.

## 1 Introduction

Plaque psoriasis (PSO) is a chronic immune-mediated skin disease affecting the functional capacity and overall wellbeing of patients (1–3). PSO poses a considerable burden both to patients and society (1, 4–7), with increased productivity losses (8) and elevated healthcare resource utilization (HCRU) (9). Effective management of PSO should focus on minimizing physical and psychological impairments while maintaining the patient’s ability to work, ideally through early intervention (10). Early diagnosis and treatment of PSO have been shown to improve clinical outcomes and reduce HCRU (11).

Recent advancements in therapeutic targets have significantly altered the treatment landscape for PSO over the past few decades (2, 10, 12). Positive treatment outcomes, such as high response rates and good overall patient satisfaction, favor biological therapies (13). However, there is still an unmet need in the management of PSO, which is observed in the high rates of treatment discontinuation or switching of the biologics in clinical practice (14). According to the Finnish national guidelines, patients with moderate-to-severe PSO can initiate biologic treatment as a later line of treatment after other systemic treatments have failed (15). Previously, 7.3% of Finnish patients with PSO (*n* = 41,456) were reported to have used biologics before 2018 (16), while a higher estimate (approximately 30 to 40%) was presented in a study conducted earlier in the US (17). Thus, we assumed that patients treated with biologics represent the most severe end of the disease spectrum and aimed to show that initiating biologic treatment could still improve patient outcomes, especially in terms of direct and indirect costs. We aimed to further study the discontinuation and switches in biologic treatment among patients receiving biologics. Based on our hypothesis, there are still patients undergoing sub-optimal treatments, and therefore, there is a need for new and better treatment options.

This study presents data on patients with PSO using biologic medication approved for the treatment of PSO in Finland, including adalimumab, etanercept, infliximab, brodalumab, secukinumab, ixekizumab, guselkumab, tildrakizumab-asmn, rizankizumab, and/or ustekinumab, during the study period of 2013–2022. The aim of this study was to characterize PSO patients with (the switchers) and without (the first biologic cohort) previous biologic treatment, to observe which patients initiated biologics as a first- and later-line treatment, and to assess the impact of biological therapy initiation or switching in terms of changes in HCRU and work absences. The study data were retrieved from nationwide Finnish registries, with high reliability due to mandatory recording practices, i.e., the healthcare professionals have to update the medical records, referrals, and treatment summaries within 5 days of each service event (18). This study extends previous Finnish real-world data of PSO patients’ comorbidities, medication use (16), and HCRU (19), which were presented until 2018.

## 2 Materials and methods

### 2.1 Study population and design

**Figure 1:**
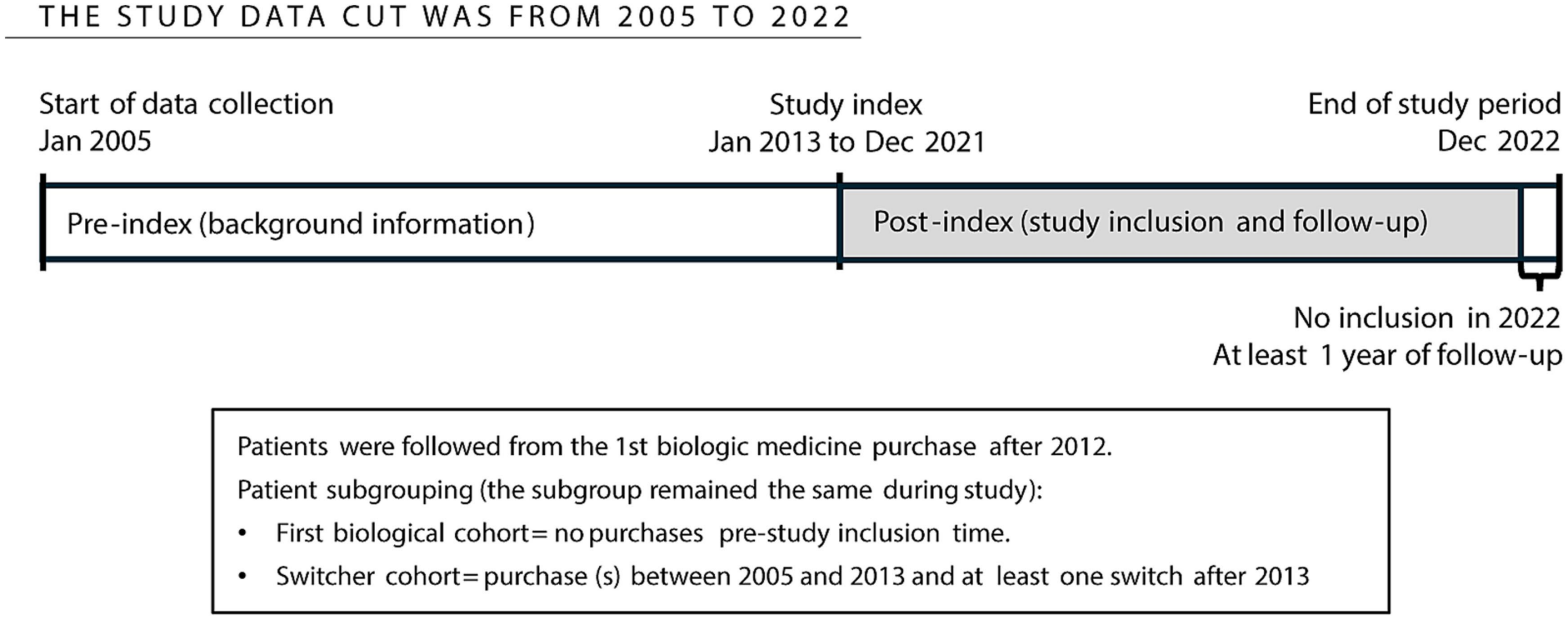
Study design and timeline.

This is a longitudinal retrospective registry-based study utilizing electronic medical records linked from four nationwide social and healthcare registry controllers in Finland (Figure 1). As Finland has a primarily taxation-funded healthcare system, the Finnish registries cover all permanent residents in the country (18, 20), which was approximately 5.6 million in 2019. The study was conducted with permissions from Findata and Statistics Finland (data permit no. THL/3560/14.02.00/2022 and TK/2979/07.03.00/2022) based on the Act on the Secondary Use of Health and Social Data (Finland’s Ministry of Justice 552/2019). Therefore, no informed consent from the patients was required.

The data cut-off of this study was from 1 January 2005 to 31 December 2022 (Figure 1). The data collected before 2013 served as background information. However, the inclusion period was from 1 January 2013, as the Finnish Institute of Health and Welfare (THL) primary care diagnoses were available only from 2012. The inclusion period from 1 January 2013 to 31 December 2021 allowed for a minimum baseline and follow-up period of 1 year for the patients.

### 2.2 Patient cohort inclusion and stratification

The data pool for study cohort formation is based on recorded International Classification of Diseases, Tenth Revision (ICD-10) -code diagnoses. Diagnosis records of patients with PSO (L40.0–40.5, L40.8–40.9), with or without psoriatic arthritis (PsA; M07.0–7.3, M13.8), obtained from secondary healthcare were included. Each patient’s diagnosis was further validated when entering the study (index = first biologic medication purchase) utilizing the granted reimbursement code 319 for injected biologic treatment for PSO and 313 or 281 for PsA. Patients with recorded reimbursement numbers for both PSO and PsA or a reimbursement number for one indication and a recorded diagnosis for the other were characterized as patients with PSO having concomitant PsA, which did not lead to an exclusion from the study. The study based on the PsA patient population will be published separately.

Patients with the granted reimbursement code and reimbursed injected biologic medication purchases (Tumor Necrosis Factor alpha (TNFα) [adalimumab, etanercept, or infliximab], Interleukin (IL) IL-12/23 [ustekinumab], IL-17 [secukinumab, brodalumab, or ixekizumab], and IL-23 [guselkumab, tildrakizumab-asmn, or risankizumab] inhibitors) during the study period were included in the study. Patients with PSO were stratified into sub-cohorts: *switchers* (biologic medication purchases before 2013 and at least one switch after 31 December 2012) and *first biological* cohort (no biologic medication purchases before 2013 and at least one purchase after 31 December 2012). The follow-up of the first biological cohort patients started at the time of the first recorded purchase of the biologic medication during the study period (2013–2021), while the switchers were included at the date of first switch after 2013. Switch was defined as a purchase of any of the aforementioned medications previously not purchased by the patient. The number of switches and the proportion of patients with the corresponding number of switches before the study index (1 January 2013) were reported. A 1:1 age- and sex-matched reference cohort was formed from PSO patients undergoing only systemic conventional therapy (Table 1). The 1:1 matched reference cohort was used only in the difference-in-difference analyses (HCRU) to account for and adjust for possible larger temporal structural changes (including but not limited to the effect of coronavirus disease 2019 (COVID-19) and structural changes in the Finnish healthcare system) in an unadjusted pre-post setting. The start of follow-up of the reference cohort patient was the date the corresponding patient entered the study. The reference cohort was utilized in analyses of sick leaves and disability pensions. The study did not capture switchers who had started biologic treatment before 1 January 2013 and had no switch by 31 December 2021; thus, the study cohort does not reflect the number of bio-experienced patients in clinical practice. The stratification remained the same throughout the study (intention to treat), where the first biological patient with several biologic medication purchases after inclusion was considered the first biologic cohort in this study.

**Table 1:** Demographics and clinical characteristics.

Characteristic		Patients with psoriasis		Patients with psoriasis (PSO) and psoriatic arthritis (PsA) ͣ	
		Switchers (*n* = 345)	First biological cohort (*n* = 2,092)	Switchers (*n* = 133)	First biological cohort (*n* = 402)
Age	Mean (SD)	53.61 (12.65)	49.57 (14.58)	53.07 (12.08)	51.05 (14.29)
	Median (IQR)	53.81 (44.84, 63.20)	49.78 (38.61, 60.26)	53.68 (45.28, 61.33)	51.34 (40.78, 61.11)
Male, *n* (%)		230 (66.67)	1,407 (67.26)	80 (60.15)	266 (66.17)
First biologic medication initiated during study time, *n* (%)*	etanercept (L04AB01)	13 (3.77)	73 (3.49)	7 (5.26)	19 (4.73)
	adalimumab (L04AB04)	42 (12.17)	351 (16.78)	20 (15.04)	114 (28.36)
	ustekinumab (L04AC05)	>113 (>32.8)	500 (23.90)	43 (32.33)	66 (16.42)
	secukinumab (L04AC10)	101 (29.28)	429 (20.51)	>45 (>33.8)	>112 (27.9)
	brodalumab (L04AC12)	<5 (<1.4)	21 (1.00)	0 (0)	<5 (<1.2)
	ixekizumab (L04AC13)	20 (5.80)	166 (7.93)	6 (4.51)	39 (9.70)
	guselkumab (L04AC16)	37 (10.72)	330 (15.77)	7 (5.26)	30 (7.46)
	risankizumab (L04AC18)	16 (4.64)	222 (10.61)	<5 (<3.9)	15 (3.73)
Follow-up time, years	Mean (SD)	6.03 (2.56)	4.70 (2.56)	6.25 (2.47)	4.96 (2.54)
	Median (IQR)	6.60 (4.06, 8.07)	4.30 (2.38, 6.74)	6.70 (4.70, 8.19)	4.75 (2.81, 6.89)
The number of biologic medication treatment lines before study time, *n* (%)	0	0 (0)	2,092 (100)	0 (0)	402 (100.00)
	1	> 205 (> 59.4)	0 (0)	>70 (>52.6)	0 (0)
	2	116 (33.62)	0 (0)	51 (38.35)	0 (0)
	3	21 (6.09)	0 (0)	9 (6.77)	0 (0)
	4	<5 (<1.4)	0 (0)	<5 (<3.8)	0 (0)
Charlson comorbidity Index (CCI), *n* (%)	0	293 (84.93)	1,915 (91.54)	111 (83.46)	361 (89.80)
	1–11	52 (15.07)	177 (8.46)	22 (16.54)	41 (10.20)
Socioeconomic status, *n* (%)	Self-employed	35 (10.14)	170 (8.13)	11 (8.72)	27 (6.72)
	Upper-level employee	34 (9.86)	251 (12.00)	17 (12.78)	33 (8.21)
	Lower-level employee	63 (18.26)	392 (18.74)	26 (19.55)	71 (17.66)
	Manual worker	48 (13.91)	433 (20.70)	13 (9.77)	82 (20.40)
	Student	6 (1.74)	63 (3.01)	<5 (<3.8)	15 (3.73)
	Pensioner	130 (37.68)	551 (26.34)	51 (38.35)	125 (31.09)
	Long-term unemployed	21 (6.09)	173 (8.27)	9 (6.77)	31 (8.71)
	Unknown	8 (2.32)	59 (2.87)	5 (2.5)	18 (4.48)
Education level, *n* (%)	Vocational school	>147 (>54.6)	1,033 (60.20)	>80 (>48.2)	>204 (>62.2)
	Bachelor’s degree	40 (14.87)	234 (13.64)	29 (17)	43 (13.11)
	Other (e.g., business school, nurse, technician)	56 (20.82)	275 (16.03)	42 (25)	54 (16.46)
	Master’s degree	19 (7.06)	156 (9.09)	10 (6.0)	24 (7.32)
	Doctoral or licentiate’s degree	<5 (<1.9)	18 (1.05)	<5 (<3)	<5 (<1.5)
	Missing	76 (22.03)	376 (17.02)	37 (18.2)	74 (18.41)
The most common concomitant medications (ATC-code) *n* (%)	Diabetes medications (A10)	72 (20.87)	313 (14.96)	27 (20.30)	60 (14.93)
	Antithrombotic agents (B01)	29 (8.41)	144 (6.88)	(6.77)	35 (8.71)
	Antihypertensives (C02)	<5 (<1.4)	19 (0.91)	<5 (<3.8)	<5 (<1.2)
	Diuretics (C03)	31 (8.99)	124 (5.93)	12 (9.02)	29 (7.21)
	Calcium channel blockers (C08)	67 (19.42)	271 (12.95)	25 (18.80)	59 (14.68)
	Agents acting on the renin-angiotensin system (C09)	140 (40.58)	625 (29.88)	44 (33.08)	135 (33.58)
	Lipid modifying agents (C10)	75 (21.74)	416 (19.89)	27 (20.30)	80 (19.90)
	Dermatologicals (D*)	279 (80.87)	1,838 (87.86)	111 (83.46)	353 (87.81)
	Corticosteroids for systemic use (H02)	42 (12.17)	217 (10.37)	26 (9.55)	61 (15.17)
	Antineoplastic and immunomodulating agents (L04), excluding the studied biologics	103 (29.86)	1,506 (71.99)	52 (39.10)	294 (73.13)
	Musculoskeletal system (M*) medications	191 (55.36)	910 (43.50)	83 (62.41)	223 (44.47)
	Psycholeptics (N05)	57 (16.52)	301 (14.39)	25 (18.80)	59 (14.68)
	Psychoanaleptics (N06)	61 (17.68)	318 (15.20)	30 (22.56)	74 (18.41)

Due to anonymity guidelines by the Finnish Central Authority, Findata, small patient groups (<5 patients) have not been reported in detail. Additionally, if only one group of size <5 per categorical variable was reported, the exact size of the largest patient group was masked (> symbol used); IQR = interquartile range; ͣ= recorded reimbursement numbers for both psoriasis and psoriatic arthritis or reimbursement number(s) for one indication and a recorded diagnosis for the other were characterized as patients having both diagnoses; ** = Infliximab has not been included in this table because of a small number of patients using the medication; rizankizumab not included in this table because it was not reimbursed/used in Finland during the study period, * = any symbol.

### 2.3 Sick leaves and disability pensions

Sick leave periods of a minimum of 10 days were covered. The cumulative mean number of absenteeism days, sick leave days, and disability pension days per patient was presented 36 months before and after entering the study. The age cap of 65 years was applied when censoring patients. The proportion of patients aged <65 on disability pension 36 months before, after, and when entering the study was reported.

### 2.4 Healthcare resource utilization

Healthcare resource utilization was characterized per patient year: 1 year before and after the start of follow-up and until the end of follow-up, death, or the end of the study period. Reasons for sick leave and HCRU were recorded as ICD-10 codes at the three-digit level (e.g., L40). The eight other sub-codes of L40 describing the PSO diagnosis in more detail were not available. As the number of patients with detailed reasons for disability pension was small, the three-digit level L40 was used. Thus, PSO/PsA was used in this study to label disease-related sick leaves, disability pensions, and HCRU. In this study, we accounted for absences due to sick leaves and pensions specifically related to PSO/PsA. HCRU is reported in a manner that distinguishes between absences due to disease-related causes, other causes, and total absences.

### 2.5 Statistical methods

Patients were characterized when entering the study using descriptive methods (median and range for continuous variables and frequencies and proportions with the 95% confidence intervals (CI) for categorical variables). Charlson comorbidity index (CCI) was defined using a modified version of the method (21). The purchased concomitant medications until the time of entering the study were reported at the category level. The proportion of first biological patients initiating N-th biological medication was assessed in multistate time-to-event analysis, reporting the descriptive Aalen-Johansen state probabilities (of the patient being on 1st, 2nd, 3rd, and 4th or more biologic medication). State transition (biological switch) was defined as the time of first purchase of a biologic medication, the time of purchase previously not purchased, or death, whichever occurred first. Each contact unit’s cost was assigned based on the contact type and specialty and the education level of the attending healthcare professional, according to Mäklin et al. (22). The costs were scaled to represent the 2023 cost level in euros (€) with an inflation correction coefficient of 1.16788. Change in HCRU was assessed using both descriptive mean cumulative functions, which describe the accumulation of costs as a function of time among patients initiating/switching biologic treatment, and using difference-in-difference analyses assessing the HCRU consumption rate 1-year pre- vs. 1-year post-index/switch date, controlled by the matched controls, who were patients receiving conventional therapy (acitretin, ciclosporin, apremilast, or dimethyl fumarate).

Statistical analyses were performed using R language version 4.0.5, using only existing data. Missing values were not imputed. The proportion of missing values was reported when applicable. The significance level was set to the conventional 0.05 for any statistical tests performed, and there was no correction for multiple testing.

## 3 Results

**Figure 2:**
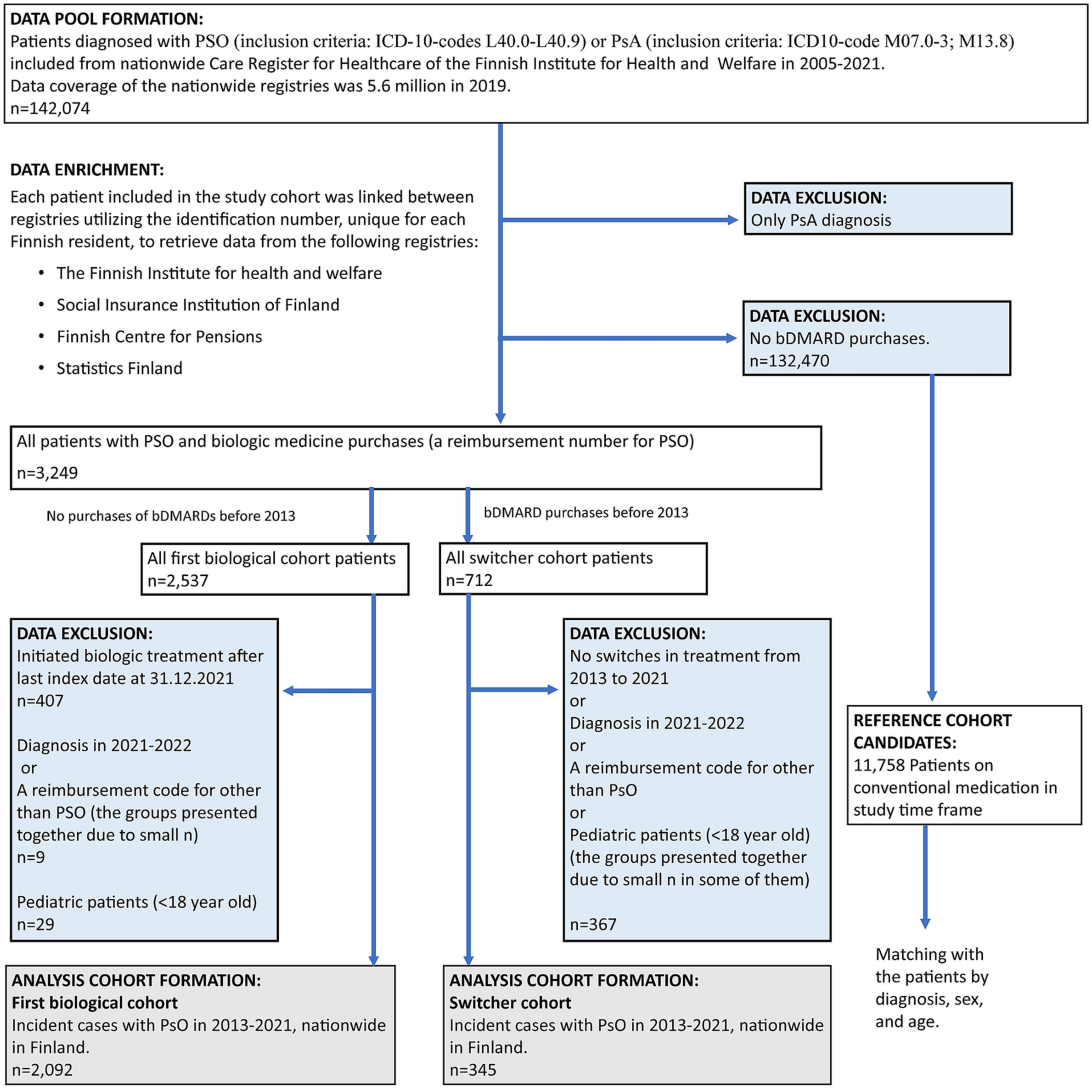
Flowchart of data formation. Excluded data (blue boxes) and analyzed cohorts (gray boxes). PSO, psoriasis; PsA, psoriatic arthritis.

A total of 2,437 patients with both PSO and biologic drug purchases were included in this study (Figure 2). Of these, 14.2% (*n* = 345) were classified as switchers—patients with biologic medication purchases before 2013 and at least one medication switch during the study period (up to the end of follow-up on 31 December 2022)—and 85.8% (*n* = 2,092) were analyzed as part of the first biological cohort (no biologic medication purchases prior to 2013) (Table 1). Among the total number of patients, 22.0% (*n* = 535) had concomitant PsA.

### 3.1 Patient characteristics

The median age of switchers was 53.8 years [interquartile range (IQR): 44.8–63.2], while the first biological cohort had a median age of 49.8 years (IQR: 38.6–60.3) (Table 1). Male individuals comprised 66.7% of switchers and 67.3% of the first biological cohort. The three most common biologic medications initiated by both patient cohorts during the study period were ustekinumab, secukinumab, and adalimumab. The most commonly initiated biologic treatment for switchers with concomitant PsA was secukinumab (>33.8%, *n* > 45), compared to 27.9% (*n* > 112) in the first biological cohort. For the first biological cohort, adalimumab was the most common treatment (28.4%, *n* = 114), while only 15.0% (*n* = 20) of switchers started with adalimumab. A substantial proportion of the switchers had experienced numerous biologic medication treatment lines before entering the study, with less than 40.6% (*n* < 142) having explored at least two biologic treatment lines. Of the switchers, 15.1% (*n* = 52) and of the first biological cohort, 8.5% (*n* = 177) had CCI > 0. The subset of patients with concomitant PsA had slightly more often elevated CCI (switchers 16.5% and first biological cohort 10.2%). Pensioner was the most common socioeconomic status (switchers = 37.7%, *n* = 130; first biological cohort = 26.3%, *n* = 551). Vocational school was the most common education level (switchers >54.6%, *n* > 147; first biological cohort = 60.2%, *n* = 1,033). The most common concomitant medications were dermatologicals, i.e., topical treatments (switchers = 80.9%, *n* = 279; first biological cohort = 87.9%, *n* = 1,838), musculoskeletal system medications, such as diclofenac, etodolac, meloxicam, and ibuprofen (switchers = 55.4%, *n* = 191; first biological cohort = 43.5%, *n* = 910), antineoplastic and immunomodulating agents, such as methotrexate, excluding the studied biologics (switchers = 29.9%, *n* = 103; first biological cohort = 72.0%, *n* = 1,506), and agents acting on the renin–angiotensin system (switchers = 40.6%, *n* = 140; first biological cohort = 29.9%, *n* = 625) category.

### 3.2 Number of biologic medications during the study period

**Figure 3:**
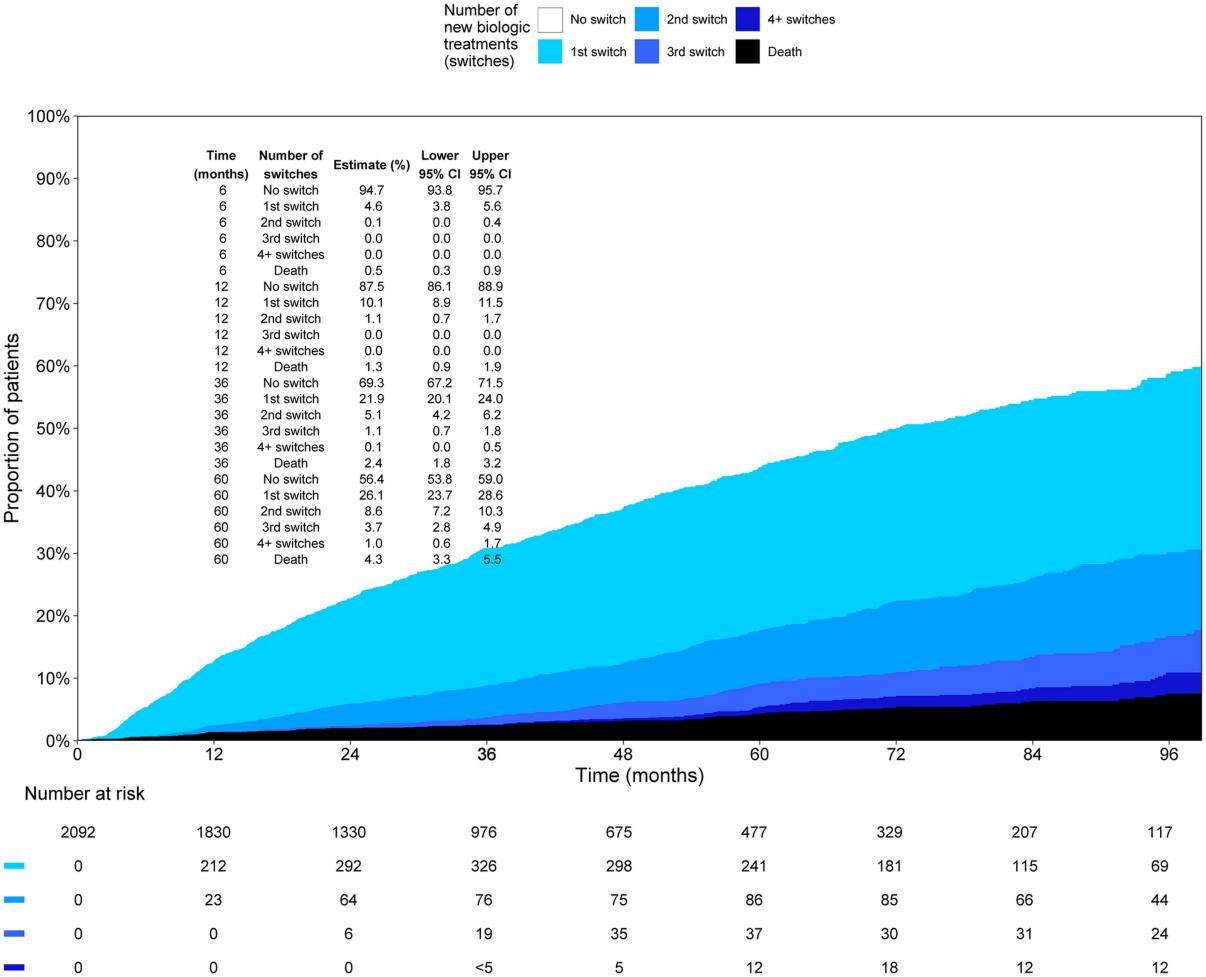
Proportion of patients on 1st, 2nd, 3rd, and 4th or more biologic medication as a function of time in the first biologic cohort.

Of the first biological cohort, 12.5% (95% CI: 11.1, 13.9) had switched their biologic medication 12 months after initiation (Figure 3). Five years after initiation, 43.6% (95% CI: 41.0, 46.2) had switched; 26.1% (95% CI: 23.7, 28.6) had one, 8.6% (95% CI: 7.2, 10.3) had two, 3.7% (95% CI: 2.8, 4.9) had three, and 1.0% (95% CI: 0.6, 1.7) had four or more switches. The median time to switch was 6 years in the first biological cohort.

### 3.3 Sick leaves and disability pensions

**Figure 4:**
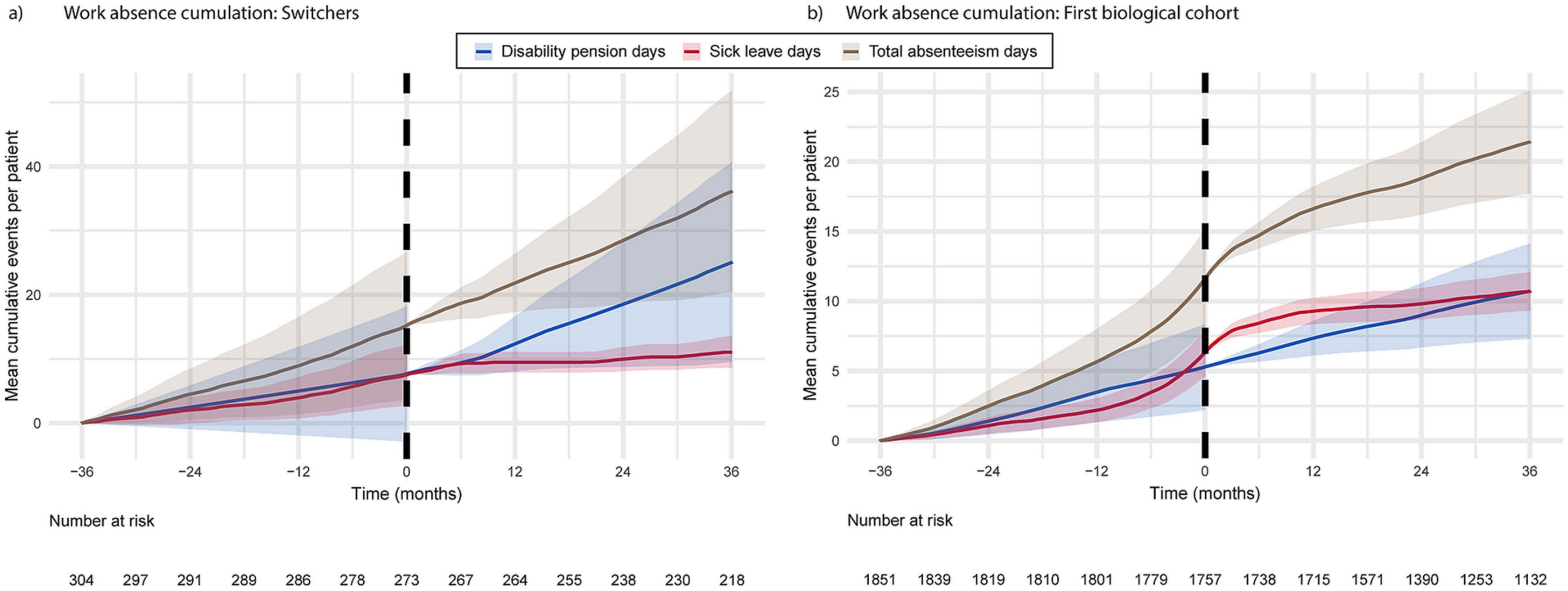
The cumulation of disease-related absence days from work for switchers **(a)** and the first biological cohort **(b)** 36 months prior to and post biologic medication initiation or switch.

The accumulation of total absences in the switchers exhibited a modest, linear increase over time. Disability pensions began to increase approximately 6 months following the switch to biologic therapy, while sick leave days decreased during the same period (Figure 4B). The accumulation of disease-related sick leaves among the first biological cohort began to increase several months before the initiation of biological treatment and decreased a few months later, whereas the accumulation of disability pensions occurred at a consistent rate throughout the study period (Figure 4A). The mean cumulative number of absence days reached 23 days per first biological cohort patient, 36 months after entering the study.

The proportion of patients on disability pension (all-cause) at index was higher in the switchers compared to the first biological cohort [7.8% (*n* = 27) and 6.6% (*n* = 138), respectively; Table 2]. In addition, registries revealed patients in the first biological cohort (*n* = 86; 4.1%) who were on disability pension at least 3 years before the biologic treatment had been initiated to manage their PSO. Out of these 86 patients, 4.6% had not yet reached retirement age (i.e., were under 65 years).

**Table 2:** The number and proportion of all psoriasis patients, as well as those under 65 years of age, receiving disability pensions were assessed 36 months before the index, at the index, and 36 months after the index.

Sub-cohort	Reason	36 months before the index		At the index		36 months after the index	
		Total *n* (%)	% of < 65	Total *n* (%)	% of < 65	Total *n* (%)	% of < 65
First biological, *n* (%)	Other	> 81 (>3.9)	>4.4	122 (5.8)	6.9	86 (6.2)	7.6
	Disease-related	< 5 (<0.2)	<0.3	16 (0.8)	0.9	14 (1)	1.2
	All-cause	86 (4.1)	4.6	138 (6.6)	7.9	100 (7.2)	8.9
Switchers, *n* (%)	Other	> 6 (>1.7)	>2.0	> 22 (>6.4)	>8.1	22 (7.7)	10.1
	Disease-related	< 5 (<1.4)	<1.6	< 5 (<1.4)	<1.8	5 (1.8)	2.3
	All-cause	11 (3.2)	3.6	27 (7.8)	9.9	27 (9.5)	12.4

Disease-related pensions are reported at the L40 level (PSO/PsA) due to the small sample size.

### 3.4 Healthcare resource utilization

The number of secondary healthcare outpatient contacts (all-cause) per year was lower 1 year after the first biological cohort patients initiated the first biologic treatment when compared to the time before biologic treatment (11.1 vs. 7.4 per patient; *p* < 0.001; Supplementary Table 1). The values compared here are Secondary healthcare inpatient days (PSO/PsA), Number of contacts, mean (95%CI) 1 year before index and 1 year after index from Supplementary Table 1. Among switchers, differences in HCRU after treatment initiation were not statistically significant. In fact, the average number of any healthcare contacts was slightly higher one year after the switch compared to one year before the switch (22.4 vs. 23.3 per patient; *p* > 0.05).

**Figure 5:**
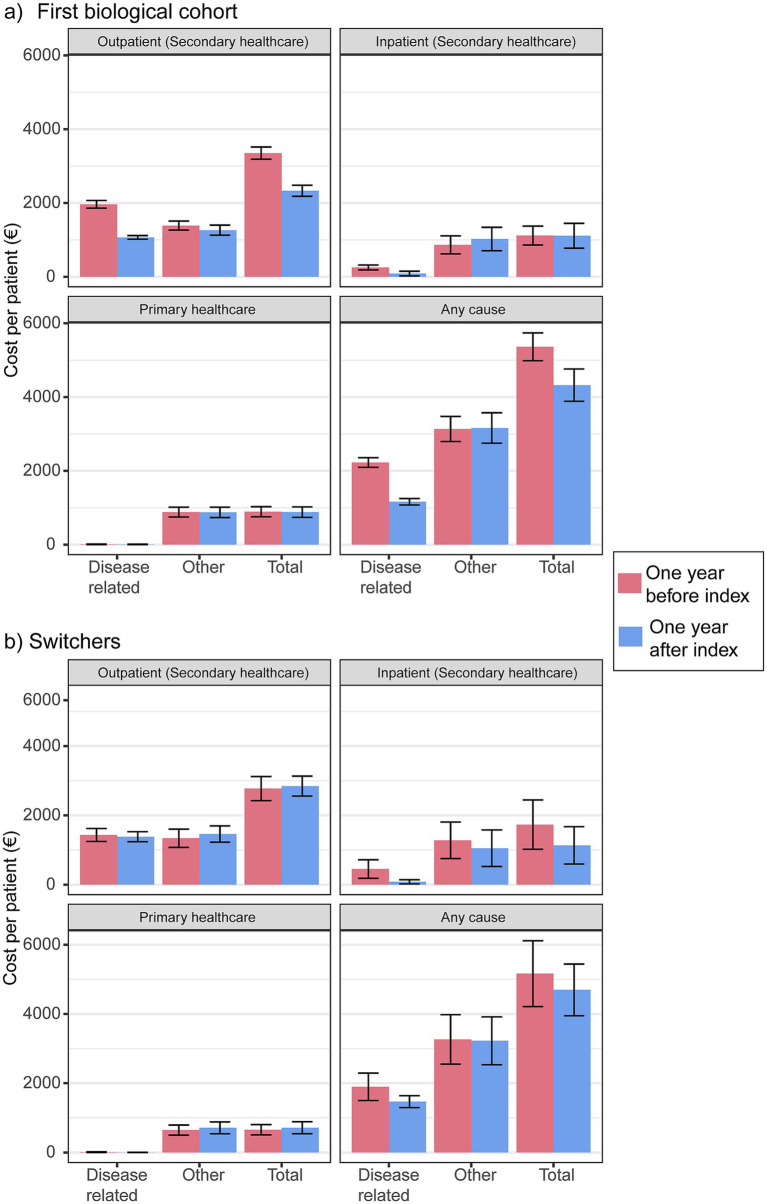
Mean cumulative healthcare resource utilization (HCRU) cost (€) by type (secondary care outpatient contacts and inpatient days, primary healthcare contacts, and any) and cause (disease-related, other, total/any) in the first biologic cohort **(a)** and the switcher **(b)** cohort.

There was a significant decrease in the disease-related any-type contact cost per year for the first biological cohort, from €2,227 (95% CI: 2,096, 2,357) to €1,161 (95% CI: 1,074, 1,248; *p* < 0.001; Figure 5, Supplementary Table 1). The mean total cost per year for the first biological cohort decreased from €5,362 (95% CI: 4,985, 5,738) to €4,323 (95% CI: 3,885, 4,761), which was not significant (*p* > 0.05). The higher number of disease-related outpatient contacts in the first biologic treatment cohort was the primary factor contributing to the difference in the annual per-patient cost reduction (€1,965 vs. €1,263; *p* < 0.001). In contrast, switchers did not show significant decreases in HCRU costs after biologic medication initiation.

## 4 Discussion

### 4.1 Key findings

This study offers an overview of PSO patients undergoing biologic treatment, examining the effects of initiating or switching biologic therapies. A total of 2,437 PSO patients in Finland, who either initiated or switched biologic medication between 2013 and 2021, were included in the analysis. Most patients with PSO did not have records of biologic medication purchases before the study period was initiated. They were sub-grouped as first biologic cohort patients without previous experience with biologics. In contrast, the switchers were the patients with biologic medication purchases before 2013 and at least one switch during the study period. The median age to initiate biologic treatment in the first biologic cohort was 50 years. Male gender was overrepresented among the patients with PSO.

In our study, over 10% of the first biological cohort switched to other biologic medications after a 1-year follow-up. Similarly, 8% of patients switched to another biologic medication in a year in the United States (23). In our study, the median time for biologic medication switch was 6 years in the first biological cohort, with a median follow-up period of 4.3 years. Previously, a much lower median time to switch was reported in Japan, 10.9 months for patients initiating first biological treatment and 9.4 months for patients with previous biological treatment (24). Good retention of the first biologic in our study could be explained by the fact that, in the Finnish healthcare system, dermatologists can choose the most suitable treatment for the patient. Though the first-line biologic treatment retention is good in Finland, many patients need multiple switches. In our study, nearly 10% of the switchers had experienced approximately 3–4 biologic medications before entering the study, demonstrating the need for highly effective drugs that could be maintained for a longer period. Our study focused on overall switching between biologic medications, meaning we captured changes between different ATC codes—such as from TNF-α inhibitors to IL-12/IL-23, IL-17, or IL-23 inhibitors—rather than switches within the same ATC code (Anatomical Therapeutic Chemical Classification System) (14), switches are common for patients with PSO; thus, patients starting to use the new biologics coming into the market are more potentially biologic switchers than patients initiating first biological treatment. Switching from one biologic to another is a strong indication of an insufficient response and is considered an unmet need in the management of PSO or a result of either primary or secondary failure of the treatment. The efficacy of treatments for PSO has shown consistent improvement over time. This is evidenced by the progressive enhancement of primary endpoints in clinical trials, evolving from 75% improvement from baseline in the Psoriasis Area and Severity Index (PASI75) responses to PASI90 and even PASI100 responses (25). Additionally, a significant number of patients achieve complete skin clearance, both in clinical trials (26–28) and real-world settings (29).

In our study, 22% of patients presented concomitant PsA, which is in line with previously reported proportions (30, 31). Our results indicate that the switchers with PSO exhibit a higher comorbidity burden, as evidenced by an elevated CCI, compared to patients initiating the first biological treatment. However, the interpretation of comorbidity findings in our study is limited by the absence of comprehensive registration of comorbidities in clinical practice. Consequently, concomitant medications served as a more reliable proxy for comorbidities in this analysis. In our study, the most common concomitant medications observed before biologic medication initiation were dermatologicals, musculoskeletal system medications, antineoplastic and immunomodulating agents (excluding the studied biologics), and agents acting on the renin–angiotensin system category. Similarly, corticosteroids, calcipotriol, methotrexate, apremilast, and cyclosporine were the most common concomitant medications in real-world patients with psoriasis in Japan (24). A notably high proportion of patients in the switcher cohort required antineoplastic and immunomodulating agents (e.g., methotrexate) concomitantly with biologic treatment for their PSO. The finding emphasizes the unmet need for more efficacious treatment options. However, in our study, the first biological cohort used antineoplastic and immunomodulating agents (excluding the studied biologics) more commonly than the switchers. The observation is in line with the treatment paradigm described in the Finnish Current Care guidelines (15) and general reimbursement criteria described by the payer, Kela (the Social Insurance Institution) (32). In Finland, the use of antineoplastic and immunomodulating agents before biologics is a mandatory step. Medications related to cardiovascular diseases and mental illnesses were also reported in our study. PSO is frequently linked with comorbidities, such as PsA, cardiovascular disease, metabolic syndrome, inflammatory bowel disease, psychiatric disorders, asthma, and malignancy (2, 6, 33). The presence of such comorbidities may impact clinical decision-making, as systemic treatments may be selected not only to manage PSO but also to address concomitant conditions (34). Although biologic treatments are highly specific and targeted toward specific modulators in the immune system, their ability to neutralize inflammation may also have a positive impact on several comorbidities (35–38), via either direct or indirect mechanisms.

Comorbidities play a critical role in both direct and indirect costs, as elevated HCRU has been previously demonstrated in PSO patients with comorbid conditions (6). Additionally, comorbidities are estimated to account for approximately two-thirds of PSO patients’ productivity losses (8). However, Mustonen et al. argued that productivity losses should be assessed on a disease-specific basis to prevent overestimation of the disease’s contribution to indirect costs (8), which was done in our study. The data revealed an increasing trend in work absence days in the first biological cohort before initiating the first medication, followed by a subsequent decrease. The absenteeism of switchers remained consistent throughout the study period. These observations suggest that the PSO patients could have benefited from an earlier initiation of biologic treatment. Reduced work capacity due to PSO may result in eligibility for disability benefits. This was evidenced by the increased accumulation of disability pension days observed after entering the study. Our data revealed that a few patients (4% of first biological cohort and 3.2% of switchers) were receiving disability pension even before any biologic treatment had been initiated to manage their disease. Disability pensions represent a substantial expense and burden for the national economy, and some could potentially be avoided through more effective patient treatment. Pensioner status was the most common socioeconomic category at index in our study, ranging from 27% in the first biological cohort to 38% among switchers with concomitant PsA. The switchers receiving disability pensions were higher than the first biological cohort. Previously, Häbel et al. reported reduced employment in Swedish patients with PSO compared to controls, despite similar socioeconomic characteristics (39).

Our study showed a significant decrease in the number of secondary healthcare outpatient contacts and disease-related inpatient days after biologic medication initiation in the first biological cohort. Previously, a remarkable decrease in the number of dermatologist contacts was reported after biologic medication initiation in Finland (19). Consistent with previous studies, the HCRU of the switchers in our study was even higher following treatment initiation (19, 24). This suggests that, when PSO persists for several years without achieving the desired treatment response, switching biologic treatments or increasing the number of switches may not be effective if the disease, particularly the underlying inflammation, has been unresolved for an extended period.

The initial assumptions were partially fulfilled. Patients who started their first biological treatment during the study period (the first biological cohort) showed a significant reduction in overall costs, although the decrease was less substantial than anticipated. Additionally, the switchers demonstrated no significant improvements in terms of direct or indirect costs. Both observations suggest that the biological treatment may have been initiated too late, and there is room for improvement in the current treatment. Additionally, we observed that a notable proportion of patients are expected to go through several switches within the first 5 years after initiating biological treatment, further supporting the assumption of a need for new and further optimized treatments.

### 4.2 Study limitations

The treatment regime of patients with psoriasis has evolved in recent years. The results of this study provide an overview of PSO management in Finland from 2013 to 2022. However, the data presented are limited to this time frame and reflect the clinical practice during that period, while certain practices may have changed since 2022. The retrospective data used in this study were originally collected for purposes other than research, which may introduce certain limitations. Consequently, some information may be missing, coding errors could be present, and diagnoses or disease severity stratification could not be validated by a specialist, potentially influencing the study’s outcomes. Patient stratification may have limitations: (1) some patients could be classified as part of the first biological cohort despite prior biologic treatment before the study period and (2) the number of switchers and first biological cohort patients may not fully reflect clinical practice, since the study only includes those who initiated or switched biologic treatment during the study period. Patients who did not initiate or switch treatment are not captured in the dataset. Regarding the first limitation, the estimated number of affected patients is minimal, as patients typically continue their prescribed treatment or switch to an alternative treatment. Long treatment gaps are rare; therefore, this limitation is unlikely to introduce significant bias. The second limitation results in smaller cohort sizes; however, these cohorts remain sufficiently large to fulfill the study’s objective of describing treatment-related changes. It is also important to note that some of the biological treatments studied have only been available in Finland for the patient group for a few years. Since the current study is purely registry-based, no patient-level data on actual medication use are available, and all conclusions are based only on purchases. Biological drugs initiated within hospitals were not accounted for. Furthermore, data on disease severity and treatment outcomes (such as Psoriasis Area and Severity Index (PASI), Body Surface Area (BSA), and Dermatology Life Quality Index (DLQI) are missing as they are not routinely recorded in a structured format in Finnish clinical practice, making it difficult to assess disease severity in the cohort patients. Therefore, treatment switches and discontinuations are determined solely based on purchases of reimbursable medications. This study covered only long-term sick leaves, specifically, periods longer than 9 days, and therefore did not account for shorter sick leaves. Furthermore, the inclusion of switchers with more than 5 previous treatment modalities may have influenced the HCRU of the patient groups. Patients with multiple failed treatment attempts are typically the most challenging to treat. In this study, HCRU after the index date was evaluated only 1 year later; however, as the number of control visits tends to decrease over time, the effect might have been pronounced 2 years after treatment initiation. As with most observational studies based on existing patient records, this study is subject to limitations related to research methods, including possible selection bias, information bias, and confounding. This study was population based and not restricted to a specific demographic; therefore, selection bias may not be a major concern. Another limitation related to the study design is that the stratification by indication was done at index, regardless of whether the patient received an additional relevant diagnosis (PSO/PsA) during follow-up. A separate sub-cohort of patients having both diagnoses was characterized separately. These limitations may impact the results; however, they are inherent to studies using medical record data and are unlikely to alter the study’s overall conclusions. In addition, the study does not reflect the status of biologics use in Finland as of 2024, since the time frame and follow-up ended in 2022. Despite these limitations, the study possesses notable strengths, including its reflection of Finnish clinical practice for patients with PSO, the inclusion of a real-world patient population, and the unbiased measurement of outcomes.

### 4.3 Conclusion

This study highlights the need for early treatment and significant unmet needs for patients with PSO. New effective treatment options are still required. To further advance the standard of care for PSO patients in Finland, patients should receive highly effective treatments that last and reduce systemic inflammation early in the disease journey, thus avoiding unnecessary sick leave periods and disability pensions. Further studies are needed to evaluate the overall benefits of early utilization of highly effective treatments.

## Data Availability

The datasets presented in this article are not readily available because this study is based on secondary use of social and healthcare registry data. Thus, data cannot be shared openly. Data can be acquired with data permission by following the guidance and application process of the registry. Requests to access the datasets should be directed to https://findata.fi/en/.
