# Supplementary material for: A population-based registry study on psoriasis-associated burden of disease in Finland
Source: Front Med (Lausanne). 2025 Jul 22;12:1605100. doi: 10.3389/fmed.2025.1605100 (PMC12322974; doi:10.3389/fmed.2025.1605100)
Supplement: Supplementary file 1 [file Table_1.docx]

**A population-based registry study on psoriasis associated burden of disease in Finland**

Ukkola-Vuoti et al., 2025

Supplementary Table 1. The number of healthcare contacts and the estimated cost one year before and after index for the patients with psoriasis by subcohorts.

| **Subcohort** | **Type** | **Reason** | **Number of contacts, mean (95%CI)** | | **Difference in difference p-value** | **Cost, mean € (95%CI)** | | **Difference in difference p-value** |
| --- | --- | --- | --- | --- | --- | --- | --- | --- |
|  |  |  | **1 y before index** | **1 y after index** |  | **1 y before index** | **1 y after index** |  |
| **First biological** | **Any** | **Other** | 21.18 (18.65, 23.7) | 20.7 (18.12, 23.28) | 0.091 | 3135 (2795, 3475) | 3162 (2750, 3574) | 0.628 |
|  |  | **PSO/PsA** | 7.81 (7.37, 8.25) | 4.15 (3.9, 4.41) | < 0.001 | 2227 (2096, 2357) | 1161 (1074, 1248) | < 0.001 |
|  |  | **Total** | 28.99 (26.37, 31.61) | 24.85 (22.22, 27.49) | < 0.001 | 5362 (4985, 5738) | 4323 (3885, 4761) | 0.172 |
|  | **Primary healthcare contacts** | **Other** | 15.81 (13.43, 18.18) | 15.45 (13.06, 17.84) | 0.009 | 882 (750, 1016) | 874 (734, 1014) | 0.332 |
|  |  | **PSO/PsA** | 0.19 (0.04, 0.33) | 0.13 (0.03, 0.23) | 0.969 | 10 (2, 18) | 7 (1, 12) | 0.980 |
|  |  | **Total** | 15.99 (13.58, 18.41) | 15.58 (13.17, 18) | 0.008 | 891 (757, 1027) | 881 (740, 1022) | 0.329 |
|  | **Secondary healthcare inpatient days** | **Other** | 1.13 (0.82, 1.45) | 1.49 (1.02, 1.97) | 0.591 | 865 (621, 1109) | 1024 (707, 1342) | 0.001 |
|  |  | **PSO/PsA** | 0.46 (0.34, 0.59) | 0.16 (0.04, 0.28) | < 0.001 | 252 (184, 320) | 87 (22, 152) | 0.042 |
|  |  | **Total** | 1.6 (1.25, 1.94) | 1.65 (1.14, 2.16) | 0.391 | 1117 (860, 1374) | 1111 (774, 1448) | 0.072 |
|  | **Secondary healthcare outpatient contacts** | **Other** | 4.04 (3.69, 4.39) | 3.55 (3.19, 3.91) | 0.297 | 1387 (1266, 1508) | 1068 (1018, 1118) | 0.604 |
|  |  | **PSO/PsA** | 7.1 (6.72, 7.47) | 3.85 (3.67, 4.02) | < 0.001 | 1965 (1862, 2068) | 1263 (1126, 1401) | < 0.001 |
|  |  | **Total** | 11.14 (10.61, 11.67) | 7.4 (6.98, 7.82) | < 0.001 | 3353 (3187, 3518) | 2331 (2180, 2482) | < 0.001 |
| **Switchers** | **Any** | **Other** | 16.29 (13.37, 19.21) | 18.22 (14.6, 21.85) | 0.645 | 3266 (2553, 3980) | 3225 (2533, 3917) | 0.778 |
|  |  | **PSO/PsA** | 6.13 (5.11, 7.15) | 5.08 (4.54, 5.63) | 0.812 | 1896 (1501, 2291) | 1469 (1298, 1641) | 0.705 |
|  |  | **Total** | 22.42 (19.18, 25.67) | 23.31 (19.53, 27.08) | 0.958 | 5162 (4213, 6112) | 4694 (3947, 5441) | 0.568 |
|  | **Primary healthcare contacts** | **Other** | 10.86 (8.48, 13.25) | 12.35 (9.26, 15.44) | 0.464 | 647 (500, 793) | 712 (539, 884) | 0.823 |
|  |  | **PSO/PsA** | 0.22 (0.02, 0.42) | 0.08 (0.02, 0.14) | 0.305 | 10 (1, 19) | 4 (1, 7) | 0.548 |
|  |  | **Total** | 11.08 (8.68, 13.49) | 12.43 (9.31, 15.54) | 0.516 | 657 (510, 793) | 716 (542, 890) | 0.838 |
|  | **Secondary healthcare inpatient days** | **Other** | 1.45 (0.92, 1.97) | 1.61 (0.73, 2.48) | 0.398 | 1280 (752, 1808) | 1052 (524, 1580) | 0.676 |
|  |  | **PSO/PsA** | 0.81 (0.31, 1.31) | 0.15 (0.04, 0.26) | 0.639 | 452 (185, 720) | 81 (21, 142) | 0.69 |
|  |  | **Total** | 2.26 (1.38, 3.14) | 1.75 (0.85, 2.65) | 0.154 | 1732 (1021, 2444) | 1133 (594, 1673) | 0.401 |
|  | **Secondary healthcare outpatient contacts** | **Other** | 3.69 (2.98, 4.4) | 4.02 (3.39, 4.65) | 0.754 | 1339 (1074, 1604) | 1383 (1238, 1528) | 0.785 |
|  |  | **PSO/PsA** | 4.99 (4.36, 5.62) | 4.83 (4.33, 5.32) | 0.535 | 1434 (1246, 1621) | 1461 (1227, 1696) | 0.675 |
|  |  | **Total** | 8.68 (7.66, 9.7) | 8.84 (8, 9.68) | 0.867 | 2773 (2425, 3122) | 2845 (2556, 3134) | 0.827 |
